# IL-18 Binding Protein–Producing Cells Attenuate Anemia in Murine Macrophage Activation Syndrome

**DOI:** 10.4049/jimmunol.2300065

**Published:** 2023-04-19

**Authors:** Mathilde Harel, Sébastien Fauteux-Daniel, Emiliana Rodriguez, Gaby Palmer, Cem Gabay

**Affiliations:** * Division of Rheumatology, Department of Medicine, University Hospitals, Geneva, Switzerland; † Department of Pathology and Immunology, Faculty of Medicine, University of Geneva, Geneva, Switzerland

## Abstract

IL-18 is a pleiotropic immunoregulatory cytokine of the IL-1 family. IL-18 has been identified as a potent IFN-γ inducer in synergy with IL-12 and IL-15 and thus as a powerful Th1 cell–polarizing cytokine. IL-18 activity is regulated by its naturally occurring soluble inhibitor IL-18 binding protein (IL-18BP), the production of which is stimulated by IFN-γ in a negative feedback loop. Circulating levels of IL-18BP are elevated, and unbound bioactive free IL-18 is thus not detectable in the circulation in physiologic conditions. However, emerging evidence indicates that the IL-18/IL-18BP balance could be dysregulated in macrophage activation syndrome (MAS), as mirrored by the presence of free IL-18 in the circulation of patients with MAS. Herein, we sought to identify IL-18BP-producing cells in a murine CpG-induced MAS model using IL-18BP knock-in tdTomato reporter mice. Endothelial cells, tissue-resident macrophages, and neutrophils appeared as major cellular sources of IL-18BP. We also identified extramedullary and medullary early erythroid progenitors as IL-18BP-producing cells in an IFN-γ-dependent manner. This finding suggests a novel regulation of IL-18 activity by erythroid precursors, which are likely involved in the prevention of the negative effects of IL-18 on erythropoiesis. Indeed, coherent in vivo and in vitro results indicate that IL-18 indirectly impairs erythropoiesis while favoring myelopoiesis and thus contributes to anemia associated with MAS and potentially with other IL-18-driven inflammatory diseases. In conclusion, IL-18BP production by endothelial cells, neutrophils, macrophages, and erythroid precursors attenuates the anemia associated with murine CpG-induced MAS.

## Key Points

- Neutrophils, macrophages, endothelial cells, and erythroid precursors produce IL-18BP.
- IL-18 indirectly impairs erythropoiesis while favoring myelopoiesis.

## Introduction

Macrophage activation syndrome (MAS) is a rare but life-threatening hyperinflammatory condition. MAS corresponds to the secondary form of hemophagocytic lymphohistiocytosis disorders. The etiology of MAS is still unclear and is probably influenced by genetic and environmental factors (1–4). MAS is secondary to inflammatory diseases, notably adult-onset Still’s disease and its childhood counterpart, systemic juvenile idiopathic arthritis; viral infections; and cancer (5). In 2018, Weiss et al. showed the presence of elevated IL-18 levels in patients with MAS (6). More important, the administration of recombinant human IL-18 binding protein (rhIL-18BP), a natural IL-18 inhibitor, successfully treated a child with recurrent episodes of severe MAS (7), indicating that IL-18 is pathogenic in this condition.

IL-18 is a potent IFN-γ-inducing cytokine belonging to the IL-1 family (8–10), the activity of which is controlled by IL-18BP (11–13). IL-18BP forms a high-affinity complex with IL-18, thereby preventing binding of the cytokine to its receptor (14–17). IL-18BP is constitutively present in high amounts in the circulation (6, 18). In a murine model of MAS induced by repeated injections of CpG, we demonstrated that IL-18BP is essential to prevent the systemic effects of the IL-18/IFN-γ axis. Dysregulation of the IL-18/IL-18BP balance, resulting in the presence of free bioactive IL-18 in the circulation, recapitulated the clinical and biological features of human MAS (18). In addition, we confirmed in vivo the existence of a negative feedback loop that controls the inflammatory effects of IL-18. Indeed, IL-18 induced the production of IFN-γ, which in turn enhanced the production of IL-18BP (19). Using bone marrow (BM) chimeric mice generated by transfer of IL-18BP knockout (KO) or wild-type (WT) BM cells into lethally irradiated IL-18BP KO or WT mice, we showed that IL-18BP is produced by both radioresistant and radiosensitive cells during CpG-induced MAS (19).

Herein, we sought to characterize the cellular sources of IL-18BP at steady state and in the CpG-induced MAS model, using IL-18BP knock-in (KI) tdTomato reporter mice. By flow cytometry and immunofluorescence, we identified macrophages, endothelial cells, and neutrophils as major IL-18BP-producing cells. In parallel, extramedullary erythroid precursors present in the spleen, as well as erythroid precursors in the BM, produced IL-18BP during CpG-induced MAS in an IFN-γ-dependent manner. To our knowledge, this finding points toward a novel mechanism by which erythroid precursors can attenuate the deleterious effects of the IL-18/IFN-γ axis. Furthermore, we showed that IL-18 exerts negative effects on erythropoiesis while favoring myelopoiesis.

## Materials and Methods

### Mice and treatments

The generation of homozygous Il18bp-tomato^ki/ki^ (KI) or Il18bp^ko/ko^ (KO) mice and their respective cohoused WT littermates is illustrated in Supplemental Fig. 1. Briefly, IL-18BP tdTomato reporter mice were created by Ingenious Targeting Laboratory (Ronkonkoma, NY). A nuclear tdTomato reporter was inserted immediately upstream of the coding region of the *Il18bp* gene. The reporter is followed by an A2 self-cleavable peptide, allowing independent production of the tdTomato and the IL-18BP proteins (Supplemental Fig. 1A). *Il18bp* gene targeting was performed by homologous recombination in iTL BF1 (C57BL/6 FLP) embryonic stem cells. Correctly targeted embryonic stem cells were microinjected into BALB/c blastocysts. Resulting chimeras with a high-percentage black coat color were mated to C57BL/6 WT mice to generate germline Neo-deleted heterozygous F1 B6-Il18bp^tm1iTL^ (Il18bp-tomato^+/ki^) mice. Genotyping was performed on total DNA extracted from ear biopsies using a three-primer PCR combining a common reverse primer (5′-GTAGCAGAGTTAGCCACATAGCTC-3′), a forward primer specific for the WT allele (5′-GACTGTTGCTTCCCAGGTAAGTCC-3′, amplified product 385 bp), and a forward primer specific for the KI allele (5′-CACCTGTTCCTGTACGGCATGGAC-3′, amplified product 153 bp) (Supplemental Fig. 1B). *Il18bp* KO mice were described previously (18, 19). Heterozygous Il18bp-tomato^+/ki^ or Il18bp^+/ko^ mice were bred in the conventional area of the animal facility of the Geneva University School of Medicine (Geneva, Switzerland) to obtain homozygous Il18bp-tomato^ki/ki^ (KI) or Il18bp^ko/ko^ (KO) mice and their respective cohoused WT littermates.

All experiments were performed on a mix of male and female, 10- to 20-wk-old, age-matched animals. To induce MAS, a class B phosphorothioate CpG 1826 oligonucleotide (5′-TCCATGACGTTCCTGACGTT-3′; Eurofins Genomics GmbH) was injected i.p. at a dose of 2.5 μg/g of body weight on days 0, 2, and 4. Following these repeated CpG injections, mice were euthanized on day 7. Assessment of clinical readouts and sample collection were described previously (18, 19). All experiments were approved by the Geneva cantonal authority for animal experimentation (licenses GE/151/17, GE/91/19, and GE/204/19).

### RT-qPCR

mRNA expression for IL-18BP was assessed in various organs in WT mice in naive conditions or following repeated injections of CpG. Total RNA from various organs was extracted using TRIzol (Life Technologies, Carlsbad, CA) and treated with RNase-free DNase (Qiagen, Germany or Promega AG). Total RNA was further cleaned up on RNeasy columns (Qiagen). cDNA was prepared from 500 ng total RNA using the SuperScript II reverse transcriptase (Life Technologies). Gene expression levels of IL-18BP and hepcidin 1 were determined by quantitative PCR (40 cycles, annealing temperature of 60°C) using the iQ SYBR Green Supermix (Bio-Rad Laboratories AG, Cressier, Switzerland). Expression levels were normalized to mRNA levels of the ribosomal large 60S subunit L32 protein (*Rpl32*), used as housekeeping gene by a comparative method (2^–ΔCt^). Non–reverse-transcribed RNA samples and water were included as negative controls. Specific pairs of primers were used to amplify *Il18bp* or *Rpl32* mRNA to generate amplicons of 234 bp or 64 bp, respectively (IL-18BP: sense primer: 5′-ACATCTGCACCTCAGACAACT-3′; antisense primer: 5′-TGGGAGGTGCTCAATGAAGGAACCA-3′, L32: sense primer: 5′-CACCAGTCAGACCGATATGTGAAAA-3′; antisense primer: 5′-TGTTGTCAATGCCTCTGGGTTT-3′). The hepcidin primers specifically amplified only hepcidin 1 (*Hamp1*) and not hepcidin 2 (*Hamp2*) cDNA (sense: 5′-TTGCGATACCAATGCAGAAGA-3′; antisense: 5′-GATGTGGCTCTAGGCTATGTT-3′; amplicon of 124 bp), because hepcidin 2 does not act on iron metabolism and anemia (20–23).

### Cell isolation procedures

Mice were anesthetized and exsanguinated by intracardiac puncture for blood collection before spleen, BM, lung, and liver were harvested. Spleens were mechanically disrupted on a 70-μm cell filter. BM was flushed from the central cavities of the femurs and tibias.

Lungs were first minced with a scalpel, and then a three-step digestion was applied at 37°C in RPMI 1640 (Life Technologies) containing dispase (0.8 mg/ml; Roche, Basel, Switzerland), collagenase P (0.2 mg/ml; Roche), and DNase I (50 µg/ml; Roche). The two first steps lasted 10 min, and the last step was stopped after complete digestion of the organ (<1 h).

The liver was digested in situ under terminal anesthesia, without prior intracardiac puncture, by cannulating the portal vein and perfusing first with a washing solution (HBSS, 0.5 mM EGTA, 25 mM HEPES, 0.1% glucose) and then with a collagenase solution (IMDM GlutaMAX, Life Technologies; 0.1% collagenase type IV, Worthington Biochemical, Lakewood, NJ; 50 µg/ml DNase I, Roche). The digested liver was dissected and mechanically disrupted in a petri dish with a scalpel. Then, the cell suspension was centrifuged twice at 68 × *g* for 2 min to separate the hepatocytes from other cell types. Then, the purification of nonhepatocyte cells was performed. Following the two low-speed centrifugations at 68 × *g*, the supernatant was centrifuged at 600 × *g* for 5 min. The pelleted cells were passed through an 8.2/17.6% OptiPrep (Axis-Shield) gradient (1400 × *g*, 30 min, without brake), and the ring of cells at the interface was recovered (24). All cell suspensions were filtered through a 70-μm filter and treated with RBC lysis buffer (155 mM ammonium chloride, 1 mM potassium bicarbonate, and 77.5 μM EDTA).

### Flow cytometric analysis

Single-cell suspensions were incubated with a rat anti-mouse CD16/CD32 Ab (Fc block, BD Pharmingen, San Diego, CA) and immunostained with mAbs specific for extracellular markers. The Ab panels are described below. Cell viability was assessed with propidium iodide or Draq7. Data were acquired on a BD LSRFortessa analyzer (BD Biosciences, San Jose, CA) and analyzed using the FlowJo analysis software (BD Biosciences). Gating strategies are illustrated in Supplemental Fig. 2.

### Immunofluorescence and cytospin staining

Mice were anesthetized and exsanguinated by intracardiac puncture before collection of spleen, liver, lung, brain, colon, heart, and kidney. Organs were fixed in 4% paraformaldehyde and equilibrated in a cryoprotective solution of 25% sucrose. Then, they were embedded in OCT and stored at −80°C. Cryostat sections were washed with PBS and Dako wash buffer (Agilent Technologies AG) before staining with the primary Abs listed below. All Abs were diluted in Dako Ab diluent (Abcam, Cambridge, UK). Primary Abs were incubated overnight at 4°C. In order to reduce autofluorescence, tissue sections were treated with the TrueVIEW Autofluorescence Quenching Kit (Vector Laboratories, Newark, CA) between incubations with the primary and secondary Abs. Sections were then washed in PBS and incubated with the appropriate secondary Ab. After being washed with Dako wash buffer and PBS, nuclei were stained with DAPI (Thermo Fisher Scientific, Waltham, MA; 1:2000 in PBS). After two successive washes with PBS, sections were mounted in FluoreGuard medium (ScyTek Laboratories, Logan, UT).

Cytospin slides of blood and BM cells were prepared using the Thermo Scientific Shandon Cytospin 4 (Shandon Scientific Ltd, Cheshire, UK) by centrifugation of 100,000 cells at 750 rpm for 7 min. For immunofluorescence staining, the cytospins were fixed in 4% paraformaldehyde for 15 min and stained as described above.

In order to be able to compare fluorescence intensities between WT and KI, as well as naive and CpG-injected mice, we sacrificed the mice to be compared at the same time, processed their organs in parallel, and performed staining simultaneously. To assess staining specificity, negative controls were performed in the absence of primary Abs (Supplemental Fig. 3).

Slides were imaged on a Zeiss Axio Imager M2 widefield microscope and analyzed with ZEN 2.6 blue software (Carl Zeiss Microscopy, Switzerland). Fluorescence was quantified using QuPath version 0.2.3 (Queen’s University).

### Abs for histological and flow cytometric analysis

For histological analysis, the primary Abs used were rabbit polyclonal anti-RFP for the detection of the tdTomato reporter protein (1:200, Abcam), rat monoclonal anti-F4/80 (1:100, clone BM8, BioLegend), goat polyclonal anti-CD31 (1:100, R&D Systems, Abingdon, UK), rat monoclonal anti-CD68 (1:100, clone FA-11, Bio-Rad Laboratories), and rat monoclonal anti-stabilin-2 (1:100, clone 34-2, MBL). Secondary Abs used were A594-conjugated donkey anti-rabbit (1:1000, Thermo Fisher), A488-conjugated donkey anti-goat (1:200, Thermo Fisher), or A488-conjugated donkey anti-rat (1:500, Thermo Fisher), as appropriate.

For flow cytometry, the standard Ab panel was composed of CD45.2-BV480 (clone 104, BD Horizon), CD3ε-FITC (clone 17A2, BioLegend), B220-BUV395 (clone RA3-6B2, BD Horizon), CD4-PerCP cyanine (Cy)5.5 (clone RM4-5, BD Pharmingen), CD4-PE Cy7 (clone RM4-5, BD Pharmingen, used only with liver cells), CD8-BV605 (clone 53-6.7, BD Horizon), NK1.1-Alexa Fluor 700 (clone PK136, BD Pharmingen), CD11b-BUV737 (clone M1/70, BD Horizon), CD11c-BV786 (clone HL3, BD Horizon), Ly6C-allophycocyanin (clone AL-21, BD Pharmingen), F4/80-BV421 (clone BM8, BioLegend), Ly6G-BV711(clone 1A8, BD Horizon), CD31-PE Cy7 (clone 390, BD Pharmingen, used only with lung cells), and CD146-PerCP Cy5.5 (clone ME-9F1, BioLegend, used only with liver cells).

In order to follow RBC maturation in vivo and in vitro, we used a specific erythrocyte differentiation Ab panel containing CD45.2-BV480 (clone 104, BD Horizon), CD11b-BUV737 (clone M1/70, BD Horizon), Ter-119-allophycocyanin (clone TER-119, eBioscience), CD71-FITC (R17217, eBioscience), and F4/80-BV421 (clone BM8, BioLegend).

To follow the expression of the IFN-γ receptor 1 (IFN-γR1) and IL-18 receptor (IL-18R)α on cells isolated from mouse BM or obtained after inducing RBC differentiation in vitro, we used an Ab panel containing CD45.2-BV480 (clone 104, BD Horizon), Ter-119-allophycocyanin (clone TER-119, eBioscience), CD71-FITC (R17217, eBioscience), CD4-PerCP Cy5.5 (clone RM4-5, BD Pharmingen), B220-BUV395 (clone RA3-6B2, BD Horizon), CD11b-BUV737 (clone M1/70, BD Horizon), F4/80-BV421 (clone BM8, BioLegend), Ly6G-BV711(clone 1A8, BD Horizon), NK1.1-Alexa Fluor 700 (clone PK136, BD Pharmingen), IL-18Rα-PE (clone P3TUNYA, Invitrogen), and IFN-γR1-BV605 (clone GR20, BD OptiBuild).

### In vitro model of RBC maturation

We used a 3-d in vitro model of RBC differentiation from BM (25). Lineage-negative (lin^−^) cells were enriched from BM by autoMACS using a lineage depletion kit (Miltenyi Biotec) following the manufacturer’s instructions. autoMACS-processed lin^−^ cells were seeded on fibronectin (2 µg/cm^2^)-coated tissue culture 12-well plates (Falcon) at a density of 1.5 × 10^5^ cells/ml/well. Cells were cultured for 24 h in IMDM-GlutaMAX containing 15% FBS, 1% BSA, 200 µg/ml holotransferrin (Sigma-Aldrich, St. Louis, MO), 10 µg/ml recombinant human insulin (Sigma-Aldrich), 10^−4^ M 2-ME, 50 U/ml penicillin G, 50 g/ml streptomycin, and 2 U/ml of erythropoietin. After 1 d of culture, the medium was replaced with IMDM-GlutaMAX containing 20% FBS; 10^−4^ M 2-ME, 50 U/ml penicillin G, and 50 g/ml streptomycin.

On day 1, cells were stimulated or not with 100 ng/ml of recombinant murine mature (rm)IL-18 (R&D Systems), 1 µg/ml of CpG, or 100 ng/ml of rmIFN-γ (R&D Systems). Blocking of IL-18 activity was performed for 30 min prior to IL-18 stimulation at 37°C with 10 μg/ml of anti-IL-18Rα Ab (clone 112624, R&D Systems). Rat IgG2a (10 μg/ml) was used as an isotype control (clone 54447, R&D Systems). Conditioned media (CM) harvested from control or IL-18-stimulated cultures were transferred onto unstimulated differentiating RBCs at day 1, 30 min after incubation with anti-IL-18Rα or isotype control. In all experiments, RBC differentiation was assessed by flow cytometry on day 3. The gating strategy is illustrated in Supplemental Fig. 2. Of note, the in vitro and in vivo characterization of erythroid precursors (from C0 to C5 in order of maturation) differs slightly. In vitro the C1 population was split into three subpopulations, C1.1, C1.2, and C1.3, whereas in vivo these subpopulations were not distinguishable.

### Laboratory tests and cytokine measurements

The levels of total IL-18BP and free IL-18 were assessed as previously described (18, 19). Mouse GM-CSF, IL-6, TNF-α, IFN-γ (eBioscience), M-CSF, CXCL-1, IL-1, and IL-33 (R&D Systems) levels were measured by ELISA according to the manufacturer’s instructions. RBCs and reticulocytes were counted using an Idexx Procyte Dx hematology analyzer (Idexx) with peripheral blood collected in EDTA-coated vials.

### Statistical analysis

Results are represented as individual values and mean ± SD. The number of included mice or cell culture wells is indicated in the figure legends, as well as the number of repetitions for each experiment. Statistical analyses were performed using GraphPad Prism 9 software (GraphPad Software, La Jolla, CA). Statistical tests used are indicated in the figure legends. *p* values <0.05 were considered significant.

## Results

### Circulating and tissue neutrophils produce IL-18BP

**FIGURE 1.**
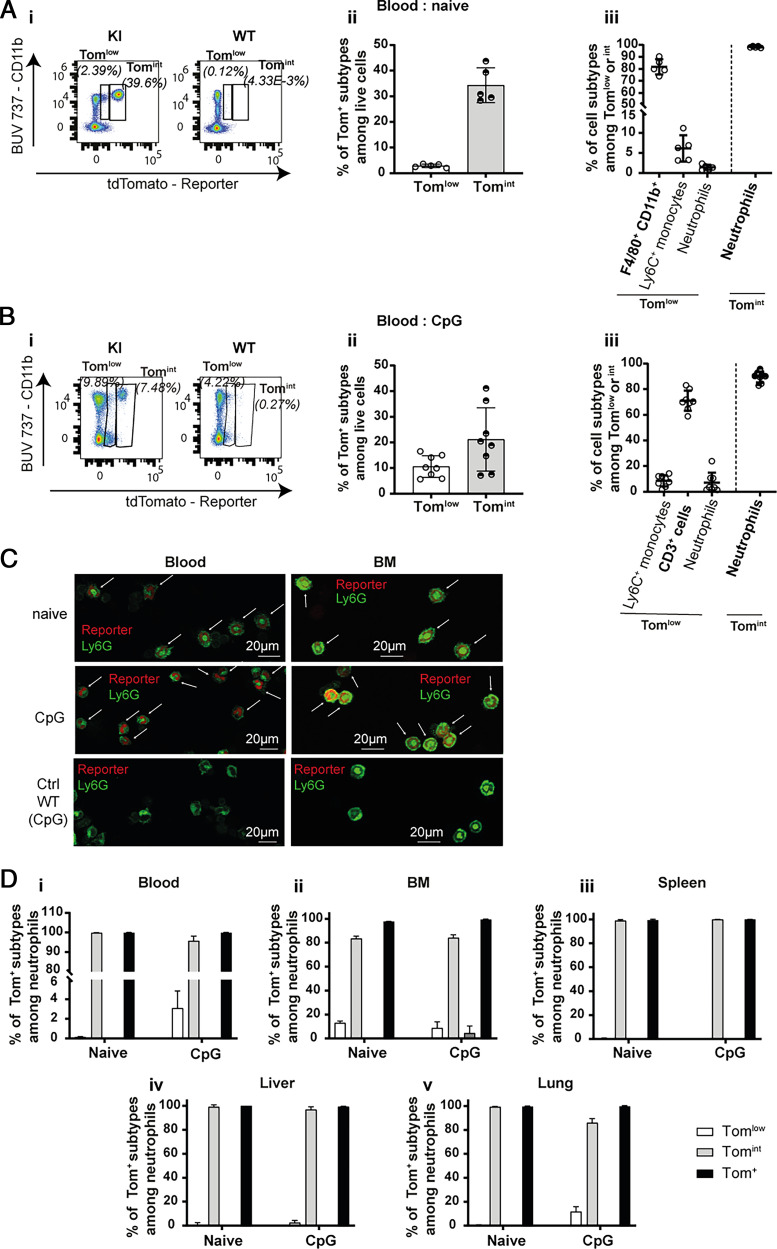
Neutrophils are IL-18BP-producing cells. Flow cytometry results obtained with blood cells of (**A**) naive (WT: *n* = 3 and KI: *n* = 5) or (**B**) CpG-injected mice (WT: *n* = 8 and KI: *n* = 8) show (A-B**i**) the presence of two populations of reporter-positive Tom^low^ and Tom^int^ cells, displaying low and intermediate reporter fluorescence intensity, respectively; (A-B**ii**) their percentage among live cells in KI mice; and (A-B**iii**) the cell types included in these Tom^low^ and Tom^int^ cell populations of KI mice. (**C**) Immunofluorescence for the tdTomato reporter (red) and for Ly6G (green) on blood (left panel) and BM (right panel) cytospin slides are shown for naive (upper panels) or CpG-injected (middle panels) KI mice and CpG-injected WT mice, shown as a negative control for tdTomato staining (lower panels). The white arrows indicate reporter and Ly6G double-positive cells. Scale bars, 20 μm. (**D**) Percentage of each reporter-positive subpopulation among neutrophils from (D**i**) blood, (D**ii**) BM, (D**iii**) spleen, (D**iv**) liver, and (D**v**) lung of naive or CpG-injected KI mice. All quantitative results are represented as mean ± SD, and each individual symbol represents one mouse. Data were generated from at least two independent experiments. Images are representative of at least two immunofluorescence analyses (randomly chosen) of three different mice per group.

The first aim of this study was to characterize the cellular sources of IL-18BP using Il18bp-tomato^ki/ki^ (KI) reporter mice (Supplemental Fig. 1A, 1B). In these mice, the nuclei of IL-18BP-producing cells display red fluorescence. By flow cytometry, two distinct tdTomato (Tom)^+^ cell populations, Tom^low^ and Tom^int^, were observed in the blood of naive and CpG-stimulated KI mice, based on their, respectively, low and intermediate reporter fluorescence intensity (Fig. 1A, 1B). Cells of WT mice did not display any fluorescence (Fig. 1Ai, 1Bi, and Supplemental Fig. 2). The frequency of Tom^int^ cells was higher than that of Tom^low^ cells in both basal and stimulated conditions (naive: 34.3% compared with 2.9% and CpG: 21.2% compared with 10.6%) (Fig. 1Aii, Bii). Independently of CpG stimulation, Ly6G^+^ cells, corresponding to neutrophils, constituted the vast majority of Tom^int^ cells (Fig. 1Aiii, Biii). Immunofluorescence anti-RFP staining on blood and BM cells of KI mice confirmed that neutrophils displayed red nuclear fluorescence (Fig. 1C). Almost all neutrophils in blood, BM, spleen, liver, and lung were Tom^int^ in both naive and CpG-stimulated conditions (Fig. 1D). Of note, Tom^low^ cells corresponded mainly to F4/80^+^ CD11b^+^ monocytes, macrophages, and/or granulocytes in naive mice, whereas CD3^+^ cells were the predominant Tom^low^ cells after CpG (Fig. 1Aiii, Biii).

### Macrophages and endothelial cells represent two important sources of tissue IL-18BP

**FIGURE 2.**
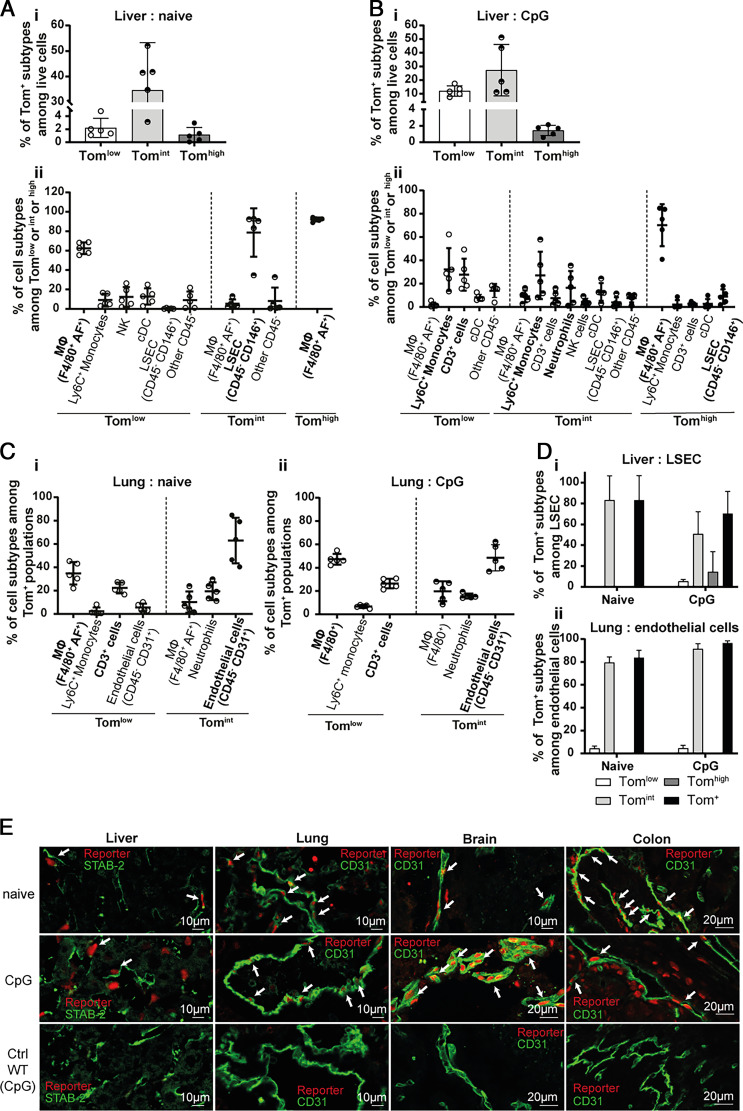
Endothelial cells are a source of IL-18BP in various organs. (**A**, **B**) Flow cytometric analysis of Tom^+^ subpopulations in liver of (A) naive (*n* = 5) and (B) CpG-injected (*n* = 5) KI mice revealing (A-B**i**) their percentage among live cells and (A-B**ii**) reporter-positive cell types. (**C**) Identification of Tom^low^ and Tom^int^ cell types in the lung of (C**i**) naive (*n* = 5) and (C**ii**) CpG-injected (*n* = 5) KI mice. (**D**) Percentage of each reporter-positive subpopulation among (D**i**) liver sinusoidal endothelial cells (LSECs) or (D**ii**) lung CD45^−^ CD31^+^ endothelial cells. All quantitative results are represented as mean ± SD, and each individual symbol represents one mouse from two independent experiments. (**E**) Immunofluorescence for the tdTomato reporter (red) and for indicated endothelial markers (green) in liver (left panels), lung (middle left panels), brain (middle right panels), and colon (right panels) of naive (upper panels) and CpG-injected KI mice (middle panels) and of CpG-injected WT mice, shown as a negative control for tdTomato staining (lower panels). The white arrows point to reporter and endothelial marker double-positive cells. Images are representative of at least two randomly chosen immunofluorescence analyses of three different mice per group.

*Il18bp* mRNA levels were highest in the liver in both naïve and CpG-stimulated mice, followed by heart, kidney, lung, colon, and spleen (Supplemental Fig. 1C). By flow cytometry, we identified three Tom^+^ cell subpopulations with low (Tom^low^), intermediate (Tom^int^), and high (Tom^high^) fluorescence intensity in the liver. The Tom^int^ subpopulation was the most abundant in both naive and CpG-stimulated conditions. The percentage of Tom^low^ cells was lower, but increased after MAS induction, whereas the percentage of Tom^high^ cells remained low without or with stimulation (Fig. 2Ai, Bi). In naive mice, F4/80^+^ autofluorescent (AF)^+^ macrophages were the most abundant cell type among Tom^low^ and Tom^high^ populations (respectively, 62.4% and 92%). CD45^−^ CD146^+^ liver sinusoidal endothelial cells were the predominant cell type in the Tom^int^ population (Fig. 2Aii). In CpG-induced MAS, many cell types were Tom^low^ and Tom^int^, including monocytes, macrophages, CD3^+^ cells, and neutrophils, but AF^+^ macrophages remained the most abundant Tom^high^ cells (Fig. 2Bii).

**FIGURE 3.**
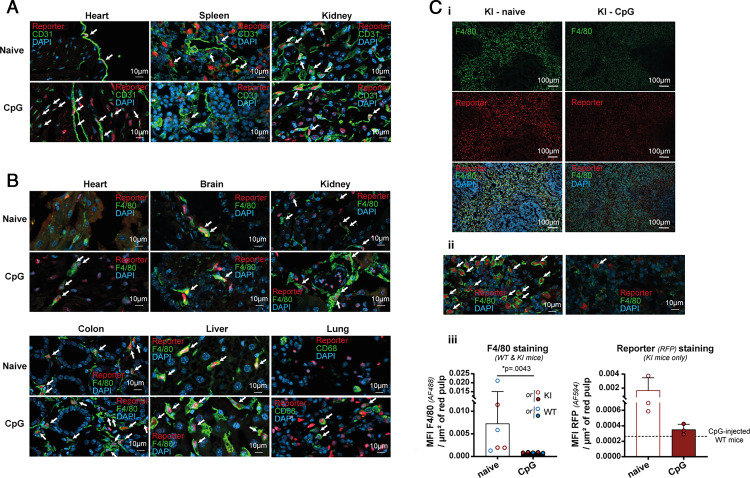
Endothelial cells and macrophages express IL-18BP. (**A**) Representative pictures of costaining for the nuclear tdTomato reporter (red) and the endothelial surface marker CD31 (green) in heart, spleen, and kidney of naïve (upper panels) or CpG-injected (lower panels) KI mice. Nuclei are labeled with DAPI. The white arrows point to cells coexpressing the tdTomato reporter and CD31. (**B**) Representative pictures of costaining for the nuclear tdTomato reporter (red) and the macrophage surface marker F4/80 (green) in heart, brain, kidney, colon, liver, and lung of naive or CpG-injected KI mice. Nuclei are labeled with DAPI. The white arrows point to cells coexpressing the tdTomato reporter and F4/80. (**Ci**) Spleen sections of naive (left panels) and CpG-treated (right panels) KI mice were stained for F4/80 (green; upper and lower panels) and for the tdTomato reporter (red; middle and lower panels). Nuclei are labeled with DAPI (lower panels). (**Cii**) Representative pictures with high magnification of costaining for the tdTomato reporter (red) and F4/80 (green) in the spleen of naive (left panel) and CpG-injected (right panel) KI mice. (**Ciii**) Quantification of the loss of fluorescence for F4/80 (left panel) and the tdTomato reporter (right panel) following CpG stimulation in the red pulp of the spleen of, respectively, WT and KI mice or KI mice only. The white arrows point to costaining. All images are representative of at least two randomly chosen immunofluorescence analyses in at least two different mice. All quantitative results are represented as mean ± SD, and each individual symbol represents one mouse. Data were generated from at least two independent experiments.

In the lung, the composition of Tom^low^ and Tom^int^ cell populations was not affected by CpG treatment. Tom^low^ cells predominantly comprised macrophages and CD3^+^ cells, and the vast majority of Tom^int^ cells were CD45^−^ CD31^+^ endothelial cells (Fig. 2C). In fact, the majority of liver sinusoidal endothelial cells in liver and CD31^+^ lung endothelial cells were tdTomato positive in both naive mice and after CpG stimulation (Fig. 2D). We further confirmed the expression of the reporter in endothelial cells and macrophages of liver, lung, brain, colon, heart, spleen, and kidney by immunofluorescence (Fig. 2E and Fig. 3A, 3B).

### Expression of IL-18BP by other cell types

Because the highest *Il18bp* mRNA levels were found in the liver (Supplemental Fig. 1C) and hepatocytes have already been described as a potential source of IL-18BP (26–28), we investigated the reporter expression in liver cells. Using a hepatocyte enrichment procedure followed by immunofluorescence and flow cytometry, we observed that hepatocytes were very weakly tdTomato positive and only following CpG injections (data not shown).

Moreover, the systematic study in different organs revealed that various immune cells were Tom^+^ to some extent (Supplemental Fig. 4). CD3^+^ cells and monocytes expressed the reporter mainly after CpG stimulations and with low intensity (Supplemental Fig. 4A, 4B). The percentage of Tom^+^ macrophages increased with CpG injections. They were predominantly Tom^low^ and Tom^int^, with the exception of liver macrophages and spleen red pulp macrophages (RPMs), which were, respectively, partially or completely Tom^high^ (Supplemental Fig. 4C). Of note, with exception of the liver, organs were harvested without whole-body perfusion. Thus, we cannot distinguish circulating cells from those present in the parenchyma; this is particularly the case for CD3^+^ lymphocytes and neutrophils in lung and spleen. Interestingly, in the spleen, CpG injections led to the disappearance of AF^+^ CD11b^−^ F4/80^high^ cells, identified as RPMs. In addition, more than half of liver NK and NKT cells were Tom^int^, but only after CpG treatment (Supplemental Fig. 4D). Finally, the proportion of Tom^+^ conventional dendritic cells increased after CpG injections (Supplemental Fig. 4Ei, 4Eii).

### Extramedullary early erythroid precursors produce IL-18BP during MAS

**FIGURE 4.**
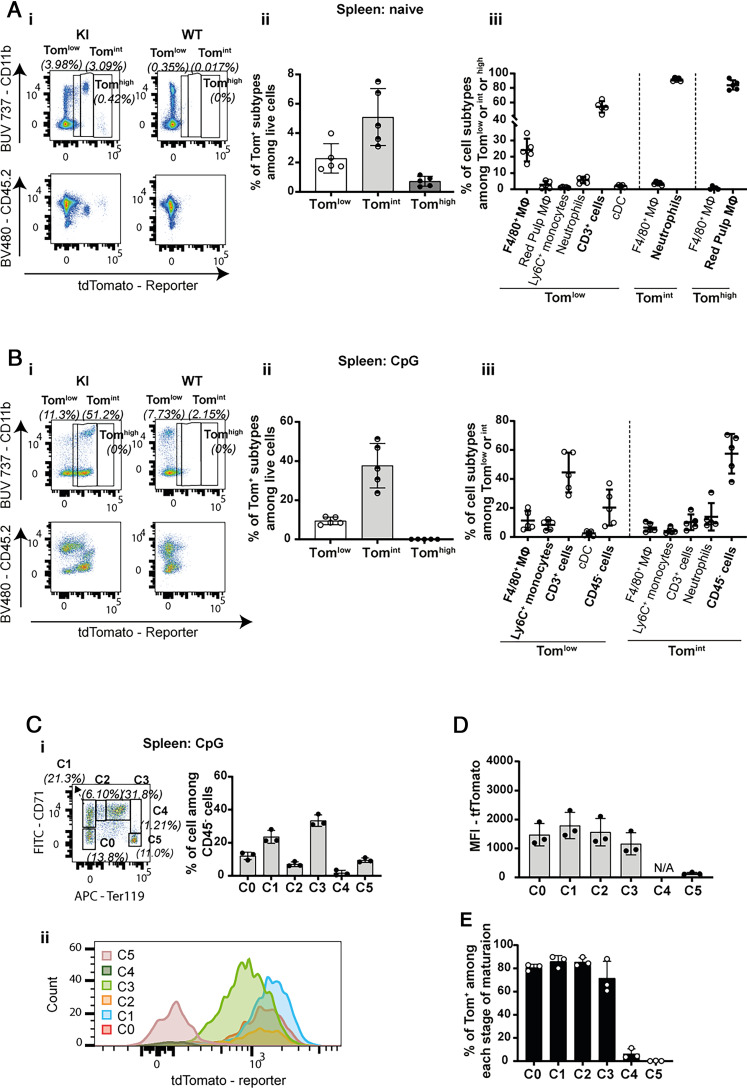
IL-18BP expression decreases with maturation in splenic erythroid progenitors. Flow cytometric analysis of tdTomato reporter expression in the spleen of (**A**) naive (WT: *n* = 3 and KI: *n* = 5) and (**B**) CpG-injected mice (WT: *n* = 5 and KI: *n* = 5) shows (A-B**i**) the presence of three subpopulations of reporter-positive cells, Tom^low^, Tom^int^, and Tom^high^, in KI mice and their respective expression of CD11b (upper panels) or CD45 (lower panels), (A-B**ii**) the percentage of Tom^+^ subtypes among live cells, and (A-B**iii**) the cell types included in these Tom^+^ subpopulations. (A, B) Results are represented as mean ± SD, and each individual symbol represents one mouse from two independent experiments. (**C**) Flow cytometric analysis of tdTomato expression by CD45^−^ splenic erythroid precursors (from C0 to C5 in the order of maturation) in CpG-injected KI mice (*n* = 3). (C**i**) Representative dot plot (left panel) and quantification (right panel, *n* = 3 KI mice) of the erythroid precursors present in spleen following CpG stimulation. (C**ii**) Representative flow cytometry histogram showing the intensity of reporter expression in different stages of maturation. (**D**) Reporter expression measured as mean fluorescence intensity (MFI) in each stage of maturation. (**E**) Percentage of reporter-positive cells in each stage of erythroid maturation. (C–E) Results are represented as mean ± SD, and each individual symbol represents one mouse. Data were generated from one experiment.

In naive mice, three small subpopulations of CD45^+^ Tom^+^ cells were identifiable in the spleen. Tom^low^ cells, representing 2.3% of live cells, corresponded to CD11b^+^ F4/80^+^ AF^−^ macrophages and CD3^+^ cells. Neutrophils were the most abundant Tom^int^ cells, which represented ∼5% of live cells. CD11b^-^ F4/80^high^ AF^+^ RPMs represented virtually the entire Tom^high^ subpopulation (Fig. 4A). Conversely, the majority of RPMs were Tom^high^ (Supplemental Fig. 4Cv). CpG stimulation dramatically modified the profile of the Tom^+^ subpopulations in the spleen (Fig. 4B). Indeed, the Tom^high^ cells disappeared, which correlated with a massive decrease of F4/80^+^ cells and Tom^+^ fluorescence in spleen sections (Fig. 3C), suggesting a loss of the RPM population after CpG stimulation.

Interestingly, a large population of CD45^−^ Tom^int^ cells was present after CpG injections, increasing the number of Tom^int^ among living cells to 38% compared with 5% in naive KI mice (Fig. 4B). These CD45^−^ cells that appeared during CpG-induced MAS reflected extramedullary erythropoiesis (28, 29). Indeed, using Abs against Ter119 and CD71, we were able to identify six distinct populations of erythroid precursors, from C0 to C5, corresponding to different stages of maturation (Fig. 4Ci). We observed that the expression of the reporter was present primarily at early stages of erythroid development (Fig. 4Cii, 4D) and lost between stages C3 and C4 of maturation (Fig. 4E).

### Bone marrow early erythroid precursors produce IL-18BP during MAS

**FIGURE 5.**
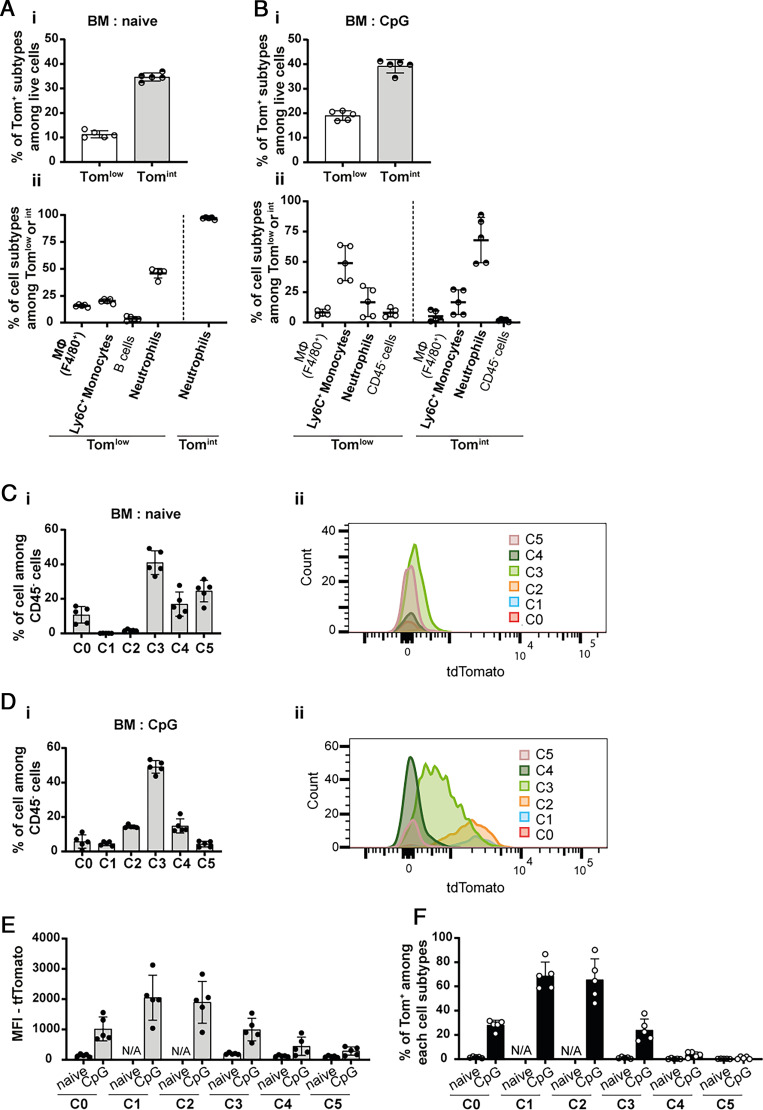
IL-18BP is expressed by erythroid precursors in BM specifically after MAS induction. Flow cytometric analysis of the reporter expression in the BM of (**A**) naive (*n* = 5) and (**B**) CpG-injected KI mice (*n* = 5) showing (A-B**i**) the percentage of Tom^+^ subtypes among live cells and (A-B**ii**) the cell types included in these Tom^+^ subpopulations. (**C**, **D**) Flow cytometric analysis of tdTomato reporter expression in the CD45^−^ medullar erythroid precursors (from C0 to C5 in the order of maturation) of naive (C, *n* = 5) and CpG-injected (D, *n* = 5) KI mice. (C-D**i**) Percentage of each erythroid precursor population among CD45^−^ cells. (C-D**ii**) Representative flow cytometry histograms showing the intensity of reporter expression in different stages of maturation. (**E**) Mean fluorescence intensity (MFI) of the reporter in each erythroid precursor cell. (**F**) Percentage of Tom^+^ cells for each stage of erythroid maturation. All quantitative results are represented as mean ± SD, and each individual symbol represents one mouse. Data were generated from two independent experiments.

In order to determine whether IL-18BP was expressed by erythroid precursors both in physiologic conditions and in the context of inflammatory responses, we analyzed the presence of Tom^+^ cells in the BM of naive and CpG-stimulated KI mice. Tom^low^ and Tom^int^ cells included macrophages, monocytes, and neutrophils, as well as monocytes and neutrophils, respectively. Tom^low^ and Tom^int^ CD45^−^ cells appeared only following CpG-induced MAS and in limited proportion (Fig. 5A, 5B). Using the same erythroid differentiation panel analysis as in the spleen, we observed that Tom^+^ cells were present solely in the C0 to C3 subpopulations, and specifically following CpG stimulation (Fig. 5C, 5D). This result was further confirmed by quantitative mean fluorescence intensity analysis (Fig. 5E). Finally, using KI mice injected with CpG, we showed that the expression of the reporter ceased in the BM between the C3 and C4 maturation stages (Fig. 5F), as during the extramedullary erythropoiesis in the spleen.

### IFN-γ-dependent production of IL-18BP by early erythroid precursors

**FIGURE 6.**
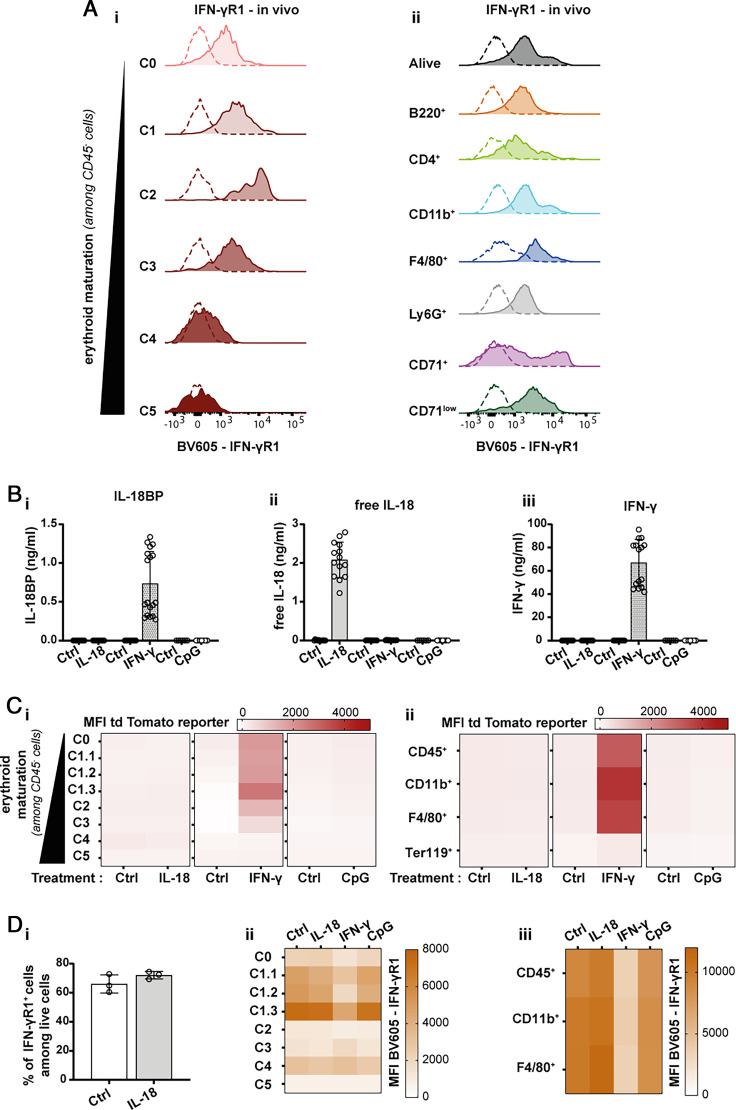
Early erythroid precursors produce IL-18BP in an IFN-γ-dependent manner. (**A**) Representative histograms for in vivo expression of IFN-γR1 by (A**i**) erythroid precursors and (A**ii**) other cell types present in the BM of naive WT mice (*n* = 4). (**B**) Assessment of (B**i**) IL-18BP, (B**ii**) free IL-18, and (B**iii**) IFN-γ levels in the supernatants of in vitro differentiated WT RBCs stimulated with rmIL-18 (100 ng/ml), rmIFN-γ (100 ng/ml), or CpG (1 µg/ml) (each individual symbol represents one culture well, *n* ≥ 6 pooled from at least two independent experiments). Results are represented as mean ± SD. The detection limits of the IL-18BP, free IL-18, and IFN-γ measurements correspond, respectively, to 0.031 ng/ml, 12.3 pg/ml, and 15.6 pg/ml. (**C**) Heat map representation of the mean fluorescence intensity (MFI) of tdTomato reporter expression (C**i**) in each erythroid precursor subtype (from C0 to C5 in the order of maturation) or (C**ii**) in other cell types following stimulation with rmIL-18 (100 ng/ml), rmIFN-γ (100 ng/ml), or CpG (1 µg/ml) of in vitro differentiated RBC cultures isolated from naive KI mice. (**D**) In vitro differentiated WT RBCs from naive WT mice were stimulated or not with rmIL-18 (100 ng/ml), rmIFN-γ (100 ng/ml), or CpG (1 µg/ml). Expression of IFN-γR1 was analyzed by flow cytometry. (D**i**) Percentage of IFN-γR1^+^ cells among live cells following rmIL-18 and rmIFN-γ stimulation. (D**ii**) Heat map representation of the MFI of IFN-γR1 expression in each erythroid precursor subtypes (from C0 to C5 in the order of maturation) or (D**iii**) in other cell types following each stimulation. The means were calculated from *n* ≥ 3 wells, and data were generated from at least two independent experiments (with the exception of the reporter MFI measurement following CpG stimulation and the in vitro expression of IFN-γR1 from one experiment).

We recently showed that the induction of IL-18BP expression during CpG-induced MAS is IFN-γ dependent (19). We thus hypothesized that the production of IL-18BP by extramedullary and BM erythrocyte precursors might also be regulated by IFN-γ during MAS. We thus first examined the expression of the IFN-γ receptor 1 (IFN-γR1) in erythroid precursors and other BM cells in vivo. Consistent with the pattern of reporter expression by erythroid precursors, only cells of stages C0, C1, C2, and C3 expressed IFN-γR1. In addition, several other BM cell types also expressed IFN-γR1, including macrophages and neutrophils (Fig. 6A).

### *In* Fig. 5Bii, how do the authors distinguish the exogenously added IL-18 from the free IL-18 produced by the cells?

In vitro, we next stimulated lin^−^ BM cells isolated from naive WT mice with rmIL-18, rmIFN-γ, or CpG during RBC maturation. We observed that IFN-γ, but neither IL-18 nor CpG, stimulated the production of IL-18BP (Fig. 6Bi). Of note, the exogenously added rmIL-18 and rmIFN-γ were detected in this assay (Fig. 6Bii, 6biii). Importantly, however, neither IL-18 nor CpG induced IFN-γ production (Fig. 6biii), suggesting that IFN-γ directly stimulates IL-18BP production.

Of note, none of these stimulations were associated with increased cell death (test of the lactate activity; data not shown). The experiment was repeated with lin^−^ BM cells of KI mice to follow the expression of the tdTomato reporter in different cell types. Consistent with the data obtained for the endogenous IL-18BP, IFN-γ, but neither IL-18 nor CpG, stimulation induced the expression of the reporter in erythroid precursors. Most important, the reporter followed the same expression pattern in in vitro erythroid precursors as previously observed in the spleen and BM of CpG-injected mice. Using the in vitro model of RBC maturation, we observed that, in addition to early erythroid precursors, other cell types, including CD45^+^ CD11b^+^ and CD45^+^ CD11b^+^ F4/80^+^ cells, contribute to IFN-γ-stimulated IL-18BP production (Fig. 6C). Consistently, the in vitro expression of IFN-γR1 followed the same pattern. Indeed, IFN-γR1 was mainly expressed by the erythroid precursors from C0 to C1.3 and myeloid cells (CD45^+^, CD45^+^ CD11b^+^, CD45^+^ CD11b^+^ F4/80^+^). Of note, the detection of IFN-γR1 decreased when cells were stimulated with IFN-γ (Fig. 6D), possibly related to receptor internalization after ligand binding and/or epitope masking.

### IL-18 impairs erythropoiesis in an in vitro model of RBC differentiation while favoring myelopoiesis

**FIGURE 7.**
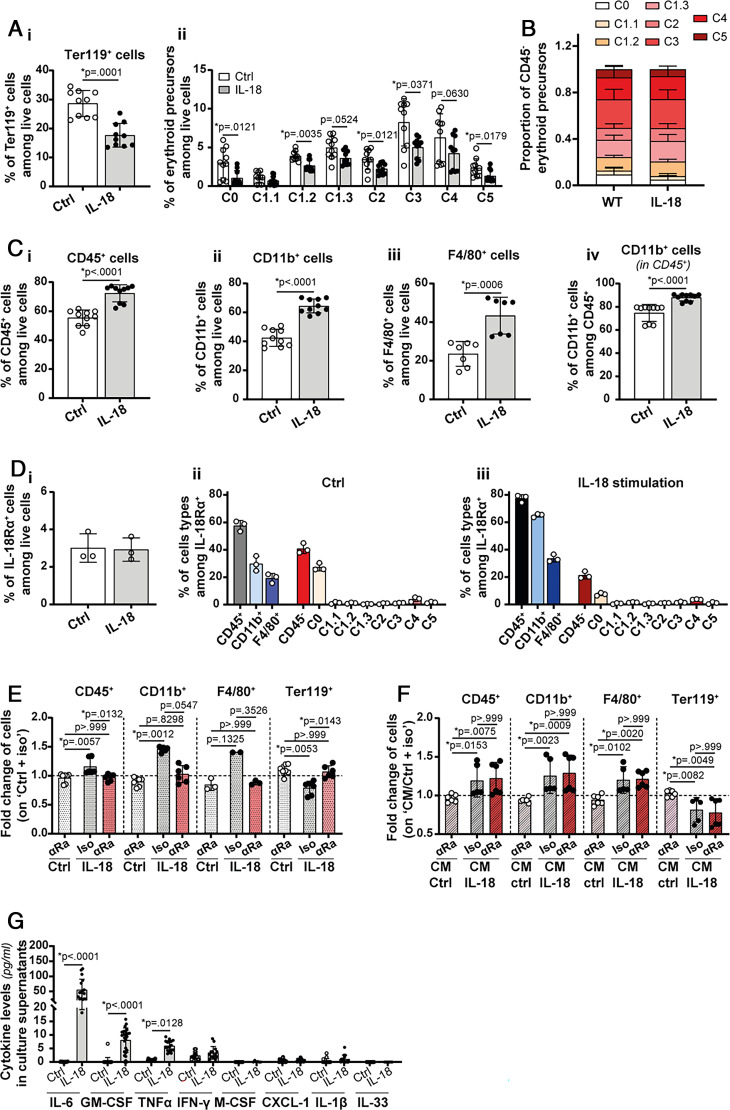
IL-18 reduces erythropoiesis and promotes myelopoiesis by an indirect mechanism. (**A**, **B**) In vitro differentiated RBCs from naive WT BM were stimulated or not with rmIL-18 (100 ng/ml). The effect of IL-18 stimulation on in vitro differentiation of RBCs from naive WT BM was assessed by evaluating (A**i**) the percentage of Ter119^+^ cells among live cells, (A**ii**) the percentage of erythroid precursors among live cells, and (B) the proportion of each subpopulation of erythroid precursors. In addition, (**C**) the effect of IL-18 stimulation on CD45^+^ populations was assessed by evaluating (C**i**) the percentage of CD45^+^ cells among live cells, (C**ii**) the percentage of CD11b^+^ cells among live cells, or (C**iv**) among CD45^+^ cells and (C**iii**) the percentage of F4/80^+^ cells among live cells. (**D**) In vitro differentiated WT RBCs from naive WT mice were stimulated or not with rmIL-18 (100 ng/ml). (D**i**) Percentage of IL-18Rα^+^ cells among live cells following rmIL-18 stimulation. (D**ii**) Identification of the cell types present among IL-18Rα^+^ cells in the control situation and (D**iii**) following rmIL-18 stimulation. (**E**) In vitro differentiated RBCs from naive WT BM were pretreated with anti-IL-18Rα (αRa) or isotype control (Iso) Abs at 10 µg/ml. Then, cells were left untreated or stimulated with 100 ng/ml of rmIL-18 (Ctrl or IL-18). The effect of the IL-18Rα blockade was assessed by studying the fold change of CD45^+^, CD11b^+^, F4/80^+^, and Ter119^+^ cells among live cells (normalization on the Ctrl + iso condition). (**F**) In vitro differentiated RBCs from naive WT BM were cultured from day 1 to day 3 in the presence of CM from unstimulated (CM/Ctrl) RBC cultures or from RBC cultures stimulated with rmIL-18 (CM/IL-18), also from day 1 to day 3. Thirty minutes before the addition of CM, cells were incubated or not with anti-IL-18Rα Abs at 10 µg/ml. Then, the fold changes of CD45^+^, CD11b^+^, F4/80^+^, and Ter119^+^ cells among live cells were assessed (normalization on the CM/Ctrl + iso condition). (**G**) IL-6, GM-CSF, TNF-α, IFN-γ, M-CSF, CXCL-1, IL-1β, and IL-33 levels were assessed in culture supernatants of RBCs differentiated in vitro in the absence or presence of rmIL-18. The respective detection limits are 3.9 pg/ml, 3.9 pg/ml, 3.9 pg/ml, 15.6 pg/ml, 7.8 pg/ml, 7.8 pg/ml, 7.8 pg/ml, and 31.25 pg/ml. Results are presented as mean ± SD, and each individual symbol represents one culture well, *n* ≥ 3 from at least two independent experiments [with the exception of the analysis of IL-18Rα expression in (D): from one experiment and the F4/80^+^ fold change in (E) and (F): *n* ≥ 2 from one experiment]. Statistical analyses were performed using Kruskal-Wallis (A, B, E, F) or Mann-Whitney tests (C, G).

In order to better understand the involvement of the IL-18/IL-18BP balance in erythroid maturation, we examined the effects of IL-18 stimulation in the in vitro model of RBC maturation. The percentage of Ter119^+^ cells and of the different erythroid precursors among live cells decreased with IL-18 stimulation (Fig. 7A). However, the relative proportions of erythroid precursor populations were not affected (Fig. 7B). Of note, the percentage of CD45^+^, CD11b^+^, and F4/80^+^ cells among live cells, as well as the percentage of CD11b^+^ among CD45^+^ cells, increased in response to IL-18 stimulation in the RBC differentiation assay in vitro (Fig. 7C).

To determine whether IL-18 acted directly on RBC maturation, we first examined the expression of IL-18Rα in the in vitro model of RBC differentiation. IL-18Rα was expressed only on a few undifferentiated C0 cells within the erythroid lineage. Conversely, the majority of IL-18Rα^+^ cells were CD45^+^ CD11b^+^ myeloid cells (Fig. 7D). Then, we blocked IL-18 signaling by pretreating cells with a blocking anti-IL-18Rα Ab. We first validated the efficacy of IL-18Rα blockade by showing that the increase of CD45^+^, CD11b^+^, and F4/80^+^ and the decrease of Ter119^+^ cells induced by IL-18 were completely abrogated by a pretreatment with anti-IL-18Rα Ab (Fig. 7E). Next, CM harvested from control or IL-18-stimulated cultures were transferred onto unstimulated differentiating RBCs. CM transferred from IL-18-treated cultures increased CD45^+^, CD11b^+^, and F4/80^+^ and decreased Ter119^+^ populations, as compared with CM harvested from control cultures. However, in this setting, inhibition of IL-18Rα was devoid of any effect (Fig. 7F), indicating that IL-18 acts on RBC differentiation through an indirect mechanism by stimulating the production of soluble mediators. Altogether, these results strongly suggest that IL-18 impairs erythropoiesis through an indirect mechanism.

The Indirect effect of IL-18 on RBC maturation cannot be mediated by IFN-γ because IL-18 stimulation did not induce IFN-γ release in the supernatant of in vitro differentiated RBC (Fig. 6Biii). However, other cytokines, such as IL-6, GM-CSF, and TNF-α, might be involved because their concentrations were significantly increased following stimulation with rmIL-18 (Fig. 7G).

### Unbalanced IL-18 activity leads to decreased RBC production in CpG-induced MAS

**FIGURE 8.**
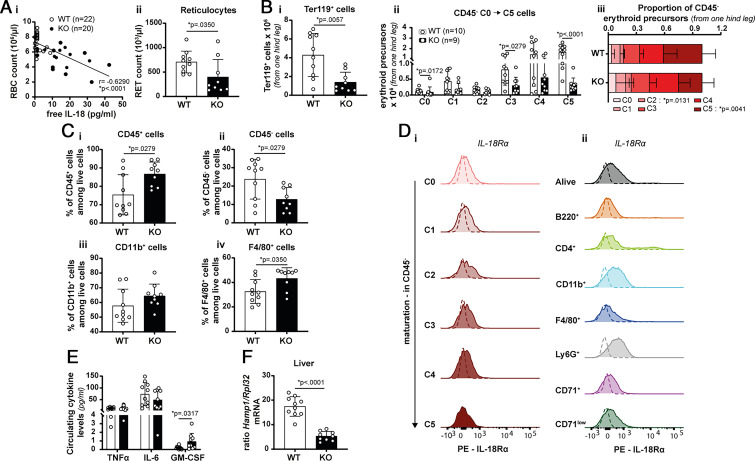
IL-18 is associated with anemia and exerts a negative effect on erythropoiesis in vivo. MAS was induced in WT and IL-18BP KO mice. Blood and organs were sampled at sacrifice (day 7). (**Ai**) The correlation between anemia and the amount of free IL-18 in the circulation is shown (WT: *n* = 22 and KO: *n* = 20, from four independent experiments). (**Aii**) Reticulocyte numbers in the blood were determined (WT: *n* = 10 and KO: *n* = 9). (**Bi**) Calculated number of Ter119^+^ cells in BM of one hind leg, based on flow cytometric analysis. (**Bii**) Absolute numbers and (**Biii**) proportion of CD45^−^ erythroid precursor populations (C0 to C5 in order of maturation) in BM of one hind leg. (**C**) Flow cytometric analysis of BM cells allowed an estimation of the percentage of (C**i**) CD45^+^, (C**ii**) CD45^−^, (C**iii**) CD11b^+^, and (C**iv**) F4/80^+^ cells (WT: *n* = 10 and KO: *n* = 9, from two independent experiments). (**D**) Representative histograms of the in vivo expression of IL-18Rα (D**i**) by erythroid precursor populations and (D**ii**) by other cell types in BM of naive WT mice (*n* = 4, from one experiment). (**E**) TNF-α, IL-6, and GM-CSF levels were assessed in the circulation of CpG-injected WT and IL-18BP KO mice, respectively (*n* = 10 and *n* = 9). (**F**) Transcript levels of *Hamp1* (encoding hepcidin 1) normalized to the housekeeping gene Rpl32 in liver. Results are represented as mean ± SD, and each individual symbol represents one mouse, *n* ≥ 7 from at least two independent experiments. Statistical analyses were performed using Mann-Whitney tests.

Elevated plasma levels of free IL-18 have been measured in IL-18BP KO mice with CpG-induced MAS (18, 19) and in patients with MAS (6). Anemia is one of the biological characteristics of MAS that can be caused by erythrophagocytosis, as well as by inflammatory responses. We observed a significant negative correlation between plasma levels of free IL-18 and RBC counts in CpG-induced MAS in IL-18BP KO mice (Fig. 8Ai). Of note, as previously shown, free IL-18 was undetectable in WT mice (18, 19). In addition, the number of reticulocytes was significantly decreased in the circulation of IL-18BP KO mice as compared with WT littermates, indicating that erythropoiesis is impaired in the presence of excessive IL-18 signaling.

We further confirmed the results obtained in vitro by following the maturation of RBCs in BM of WT and IL-18BP KO mice challenged with repeated injections of CpG. The number of Ter119^+^ cells decreased in the BM of IL-18BP KO mice in comparison with WT mice, with the highest impact on the C5 subpopulation of CD45^−^ erythroid precursors (Fig. 8Bi, 8Bii). The relative proportions of erythroid precursor populations were significantly affected (Fig. 8Biii). Furthermore, the percentage of CD45^+^, CD11b^+^, and F4/80^+^ cells increased in the BM of CpG-treated IL-18BP KO mice in comparison with their WT littermates, whereas the percentage of CD45^−^ cells was reduced (Fig. 8C). These in vivo findings also suggest that IL-18 favors myeloid cell expansion at the expense of erythropoiesis.

To identify IL-18-responsive cells in the BM in vivo, we examined the expression of IL-18Rα. The results showed that none of the erythroid precursors express IL-18Rα, suggesting that the effects of IL-18 on RBC differentiation are indirect also in vivo. Conversely, IL-18Rα was highly expressed by a small proportion of lymphocytes and to a lesser extent by neutrophils and CD11b^+^ myeloid cells (Fig. 8D). Circulating levels of GM-CSF were significantly enhanced in IL-18BP KO mice as compared with WT mice, whereas plasma levels of TNF-α and IL-6 did not differ between WT and IL-18BP KO mice following CpG stimulations (Fig. 8E). Liver hepcidin 1 mRNA levels, a downstream mediator of IL-6 involved in inflammation-associated anemia (20, 21, 23, 29–35), were decreased in IL-18BP KO as compared with WT mice (Fig. 8E). Altogether, these data suggest that the IL-6/hepcidin 1 axis and TNF-α are not involved in the anemia observed during CpG-induced MAS.

## Discussion

A variety of primary cells and cell lines of immune and nonimmune origin produce IL-18BP in vitro (28, 36–47). In this study, using IL-18BP KI tdTomato reporter mice, we demonstrated that IL-18BP is primarily produced by tissue and circulating neutrophils; resident macrophages, such as Kupffer cells and RPMs; and endothelial cells in vivo. However, other cell populations, such as T lymphocytes, dendritic cells, NK cells, and monocytes, can also participate to a lesser extent in the production of IL-18BP under inflammatory conditions. In a previous study, we observed that both radioresistant and radiosensitive cells produce IL-18BP, but that their relative contributions vary according to the organs examined (19). Accordingly, we showed that neutrophils and RBC precursors are the main IL-18BP-producing cells in the spleen, whereas endothelial cells and tissue macrophages are the main cellular source in the lung and liver.

We also showed that early erythroid precursors present in the spleen and BM produce IL-18BP during MAS in an IFN-γ-dependent manner and may therefore contribute to the prevention of the pathogenic effects of the IL-18/IFN-γ axis. Free IL-18 levels inversely correlated with blood erythrocyte counts, and BM maturation of erythroid precursors was decreased in IL-18BP KO compared with WT mice, during MAS. IL-18 inhibited RBC maturation without stimulating IFN-γ production in an in vitro RBC differentiation assay. In addition, we showed that reticulocyte counts were lower in IL-18BP KO than in WT mice, thus supporting the notion that impaired erythropoiesis is associated with excessive IL-18 signaling. Altogether, these findings suggest that IL-18 plays a pathogenic role in anemia during MAS.

The absence of IL-18Rα at the cell surface of CD45^−^ erythroid precursors suggests that IL-18 is devoid of any direct effect on erythropoiesis and thus indicates that the production of IL-18BP by erythroid precursors does not exert an autocrine protective effect. We observed that in CpG-induced MAS, the percentage of CD45^+^, CD11b^+^, and F4/80^+^ cells is increased in IL-18BP KO as compared with WT mice. Consistently, IL-18 stimulates the expansion of myeloid cells in vitro. Taken together, these findings suggest that IL-18 stimulates myelopoiesis at the expense of erythropoiesis during CpG-induced MAS.

IL-18 stimulated the production of different cytokines, including IL-6, in the in vitro RBC maturation assay. IL-6 is a pleiotropic cytokine that is involved in the occurrence of systemic manifestations, such as inflammation-associated anemia. However, despite more severe anemia following CpG injections, circulating levels of IL-6 were not increased in IL-18BP KO as compared with WT mice. Of note, IL-6 upregulates the production of hepcidin 1 by hepatocytes, which in turn inhibits the absorption of iron by the digestive tract and promotes the storage of iron by macrophages (20, 21, 23, 29–35). In consequence, the stimulation of the IL-6/hepcidin 1 axis reduces the bioavailability of iron for hemoglobin synthesis and erythropoiesis. In this study, hepcidin-1 mRNA levels were not increased, but rather significantly decreased, in IL-18BP KO compared with WT mice, further indicating that IL-6 is probably not involved in the development of anemia in CpG-induced MAS.

In vitro RBC maturation cannot recapitulate the complexity of erythropoiesis observed in vivo. Indeed, RBCs, for instance, differentiate in vitro without surrounding T and NK cells. In consequence, IFN-γ was barely detectable in culture supernatants after IL-18 stimulation, whereas IFN-γ levels are elevated in the circulation of mice with MAS, particularly in IL-18BP KO mice (18). Anemia associated with CpG injections is assumed to be mainly, but not totally, driven by IFN-γ in vivo (18, 48). Indeed, inhibition of IFN-γ signaling was not sufficient to completely rescue the severe anemia displayed by IL-18BP KO mice upon CpG injections, although the molecular signature of the IFN-γ response was drastically decreased (18).

IFN-γ is assumed to be implicated, via different mechanisms, in the occurrence of inflammatory anemia. In fact, IFN-γ promotes hemophagocytosis and, in consequence, leads to decreased RBCs in the circulation (49). In addition, IFN-γ acts as a suppressor of erythropoiesis (50–52). Indeed, IFN-γ drives anemia by inhibiting the differentiation of early erythroid progenitors through the IRF-1/PU.1 axis (53). However, neutralization of IFN-γ was not sufficient to fully rescue anemia in a model of fulminant MAS. Of interest, as in CpG-induced MAS, circulating GM-CSF levels increased in mice with fulminant MAS (54), suggesting several cytokines downstream of IL-18 are involved in myeloid proliferation and anemia.

We observed that RPMs were no longer detected in CpG-treated mice. The concomitant decrease of F4/80 staining and of tdTomato reporter expression supports the hypothesis of RPM cell death. The activation of macrophages, notably by IFN-γ, could lead to an excessive erythrophagocytosis by RPMs. Youssef et al. recently showed that an enhanced erythrophagocytosis by RPMs leads to a specific iron-dependent cell death termed “ferroptosis” (55). Of note, we previously observed the presence of erythrophagocytosis in the CpG model of MAS (18). However, although RPM cell death by ferroptosis seems highly plausible, we could also hypothesize other potential alternative cell death mechanisms, such as pyroptosis. In addition, the spleen microarchitecture is highly perturbed following CpG injections and may thus also participate in the disappearance of RPMs (56).

In conclusion, neutrophils, tissue macrophages, endothelial cells, and early erythroid precursors produce IL-18BP during CpG-induced MAS. Indeed, RBC precursors respond to IFN-γ by producing IL-18BP. Early erythroid precursors do not express IL-18Rα, but IL-18 is detrimental to RBC maturation via an indirect mechanism leading to myeloid cell expansion.

## Supplementary Material

Supplemental 1 (PDF)
