# Supplementary material for: IL-18 Binding Protein–Producing Cells Attenuate Anemia in Murine Macrophage Activation Syndrome
Source: J Immunol. 2023 Apr 19;210(11):1790–803. doi: 10.4049/jimmunol.2300065 (PMC7614543; doi:10.4049/jimmunol.2300065)
Supplement: Supplemental 1 (PDF) [file JI_2300065_Supplemental_1.pdf]

Supplemental Figure 1

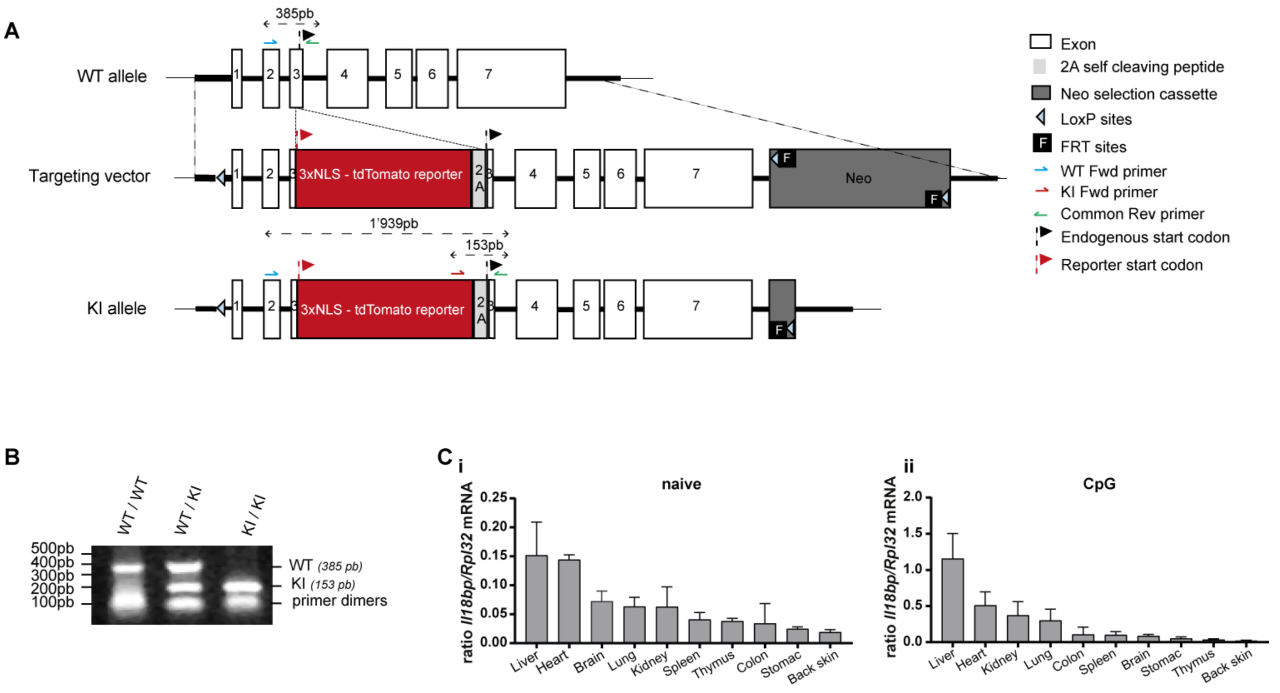

Supplemental Figure 1. Generation of Il18bp-tomato$^{ki/ki}$ (KI) mice and screening for mRNA IL-18BP expression. (A) Schematic representation of the WT allele, the targeting vector and the KI allele. A tdTomato reporter gene, carrying three nuclear localization signals (NLS), was inserted immediately upstream of the IL-18BP start codon in the first coding exon (exon 3) of the *Il18bp* gene. The nuclear tdTomato reporter and the IL-18BP coding region are separated by a sequence encoding an A2 self-cleavable peptide, allowing independent production of the reporter and the IL-18BP protein. In KI mice, the nuclei of IL-18BP producing cells display red fluorescence that can be visualized directly by flow cytometry or by histology with an anti-RFP antibody. (B) PCR genotyping of WT, heterozygous and homozygous KI mice. Primer location is shown in (A). The size of the WT and KI PCR products is indicated on the right. (C) mRNA levels of *Il18bp* were screened in various organs of naïve or CpG-stimulated WT mice. *Il18bp* expression was normalized to the housekeeping gene *Rpl32*.

Supplemental Figure 2

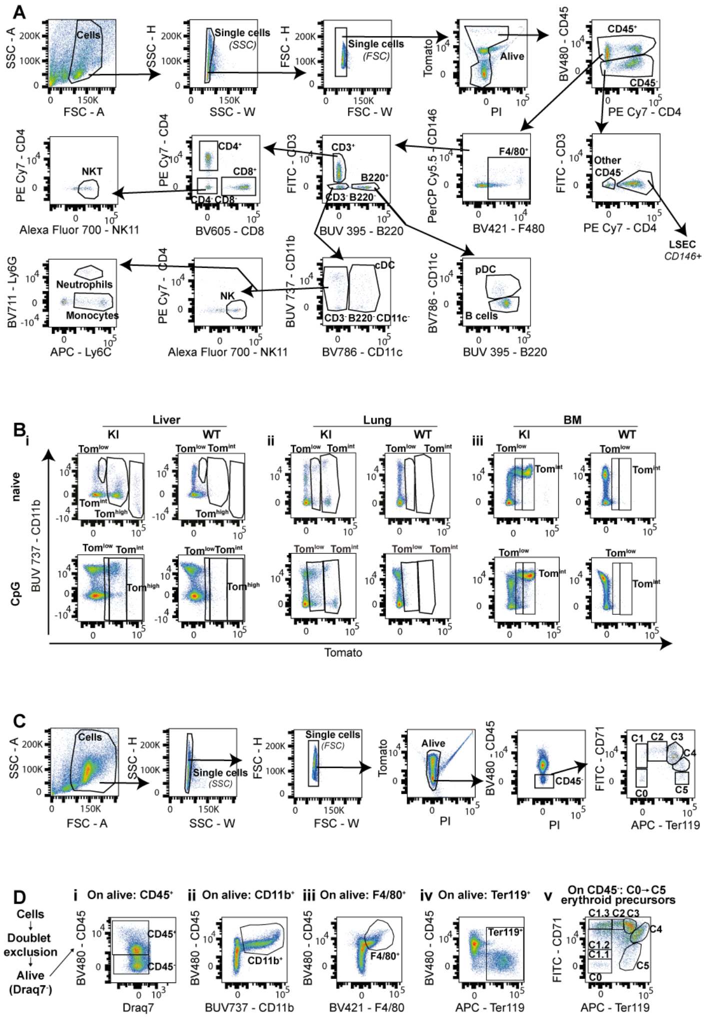

Supplemental Figure 2. Gating strategies for flow cytometry analysis. (A) Example of the gating strategy used with the standard flow cytometry antibody panel to discriminate the different cell types in the liver. (B) Representative dot plots of the subpopulations of reporter positive cells in (i) liver, (ii) lung and (iii) bone marrow (BM) from KI and WT naive mice (upper panels) and CpG-injected mice (lower panels). (C) Gating strategy used to distinguish erythroid precursors (from C0 to C5 in order of maturation) with the erythroid flow cytometry antibody panel. Spleen is shown in this example. (D) Gating strategy used in the *in vitro* model of red blood cell differentiation to separate (i) CD45^{+}, (ii) CD11b^{+}, (iii) F4/80^{+}, (iv) Ter 119^{+} cells, as well as (v) the erythroid precursors (from C0 to C5 in order of maturation).

Supplemental Figure 3

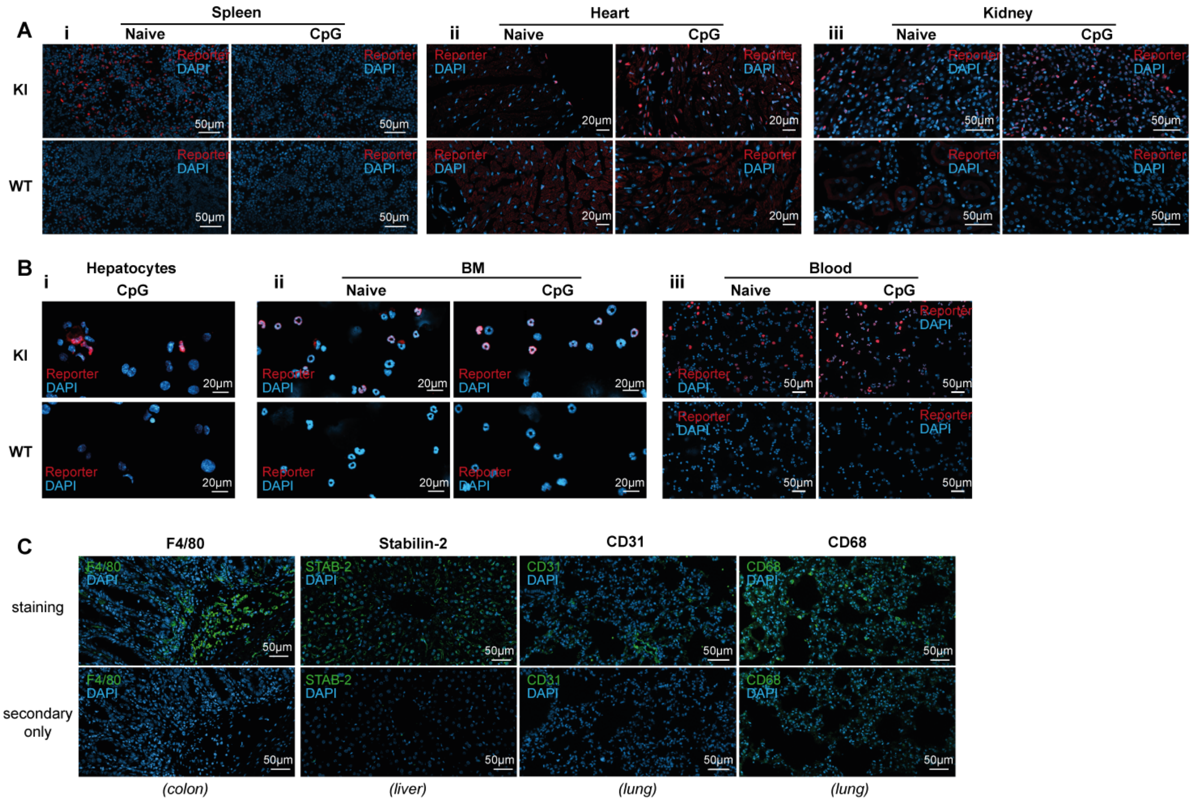

Supplemental Figure 3. Specificity controls for immunofluorescence analysis. (A-B) Specificity controls of staining for the tdTomato reporter in KI (upper panels), as compared to WT control mice (lower panels) without (naïve) or with CpG-stimulation. (A) Anti-RFP staining for the tdTomato reporter and the nucleus (DAPI) on frozen organ sections from (i) spleen, (ii) heart, (iii) kidney or (B) on cytospin slides generated with cells from (i) hepatocyte-rich fractions, (ii) bone marrow (BM) and (iii) blood. (C) Specificity controls of the antibodies used to discriminate macrophages (F4/80 or CD68), liver sinusoidal endothelial cells (STAB-2^{+} LSEC), and vascular endothelial cells (CD31). Data are representative of three randomly chosen immunofluorescence analyses in three different mice per group.

Supplemental Figure 4

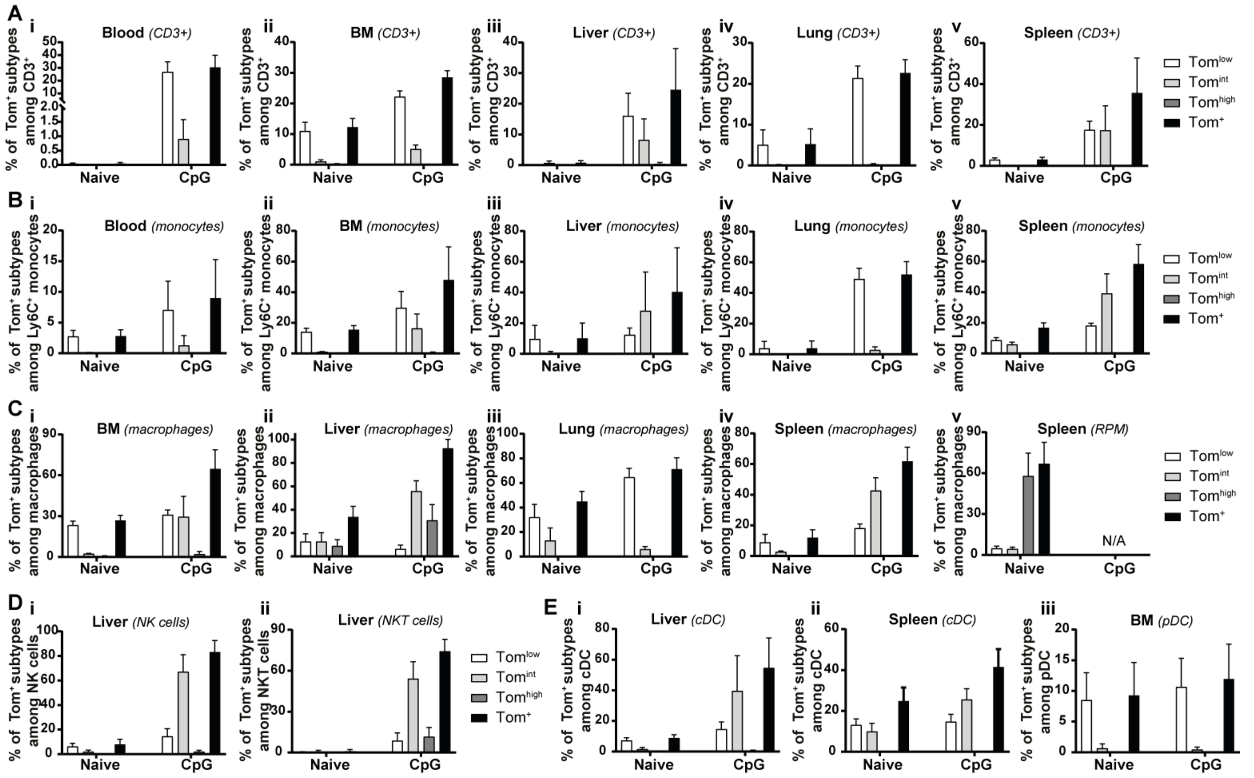

Supplemental Figure 4. Enhanced IL-18BP production by various immune cells after CpG injections *in vivo*. Percentage of Tom^{+} cells (*i.e.* sum of Tom^{low}, Tom^{int} and if existing, Tom^{high} cells) among (A) $CD3^{+}$ cells from (i) blood, (ii) BM, (iii) liver, (iv) lung and (v) spleen, in (B) monocytes from (i) blood, (ii) BM, (iii) liver, (iv) lung and (v) spleen, in (C) macrophages from (i) BM, (ii) liver, (iii) lung and (iv-v) spleen, in (Di) NK cells and (Dii) NKT cells from liver, in (E) cDC from (i) liver or (ii) spleen and in (Eiii) pDC from BM. All results are represented as mean ± SD of n≥5 naive or CpG-stimulated KI mice pooled from at least two independent experiments. N/A stands for not applicable.
